# Galectin-3 Blockade Reduces Renal Fibrosis in Two Normotensive Experimental Models of Renal Damage

**DOI:** 10.1371/journal.pone.0166272

**Published:** 2016-11-09

**Authors:** Ernesto Martinez-Martinez, Jaime Ibarrola, Laurent Calvier, Amaya Fernandez-Celis, Celine Leroy, Victoria Cachofeiro, Patrick Rossignol, Natalia Lopez-Andres

**Affiliations:** 1 Cardiovascular Translational Research. Navarrabiomed (Miguel Servet Foundation), Instituto de Investigación Sanitaria de Navarra (IdiSNA), Pamplona, Spain; 2 INSERM, Centre d’Investigations Cliniques-Plurithématique 1433, UMR 1116 Université de Lorraine, CHRU de Nancy, French-Clinical Research Infrastructure Network (F-CRIN) INI-CRCT, Nancy, France; 3 Department of Physiology, School of Medicine, Universidad Complutense, Instituto de Investigación Sanitaria Gregorio Marañón (IiSGM), Madrid, Spain; I2MC INSERM UMR U1048, FRANCE

## Abstract

**Background:**

Galectin-3 (Gal-3), a β-galactoside-binding lectin, is increased in kidney injury and its pharmacological blockade reduces renal damage in acute kidney injury, hyperaldosteronism or hypertensive nephropathy. We herein investigated the effects of pharmacological Gal-3 inhibition by modified citrus pectin (MCP) in early renal damage associated with obesity and aortic stenosis (AS).

**Results:**

Gal-3 was upregulated in kidneys from high fat diet (HFD) rats and in animals with partial occlusion of ascending aorta (AS). Urinary and plasma neutrophil gelatinase-associated lipocalin (NGAL) and urinary albumin were enhanced in HFD and AS rats. In kidney from obese rats, fibrotic markers (collagen, TFG-β), epithelial-mesenchymal transition molecules (α-smooth muscle actin, E-cadherin), inflammatory mediator (osteopontin) and kidney injury marker (kidney injury molecule-1) were modified. In kidney from AS rats, fibrotic markers (collagen, CTGF), epithelial-mesenchymal transition molecules (fibronectin, α-smooth muscle actin, β-catenin, E-cadherin) and kidney injury markers (NGAL, kidney injury molecule-1) were altered. Histologic observations of obese and AS rat kidneys revealed tubulointerstitial fibrosis. The pharmacological inhibition of Gal-3 with MCP normalized renal Gal-3 levels as well as functional, histological and molecular alterations in obese and AS rats.

**Conclusions:**

In experimental models of mild kidney damage, the increase in renal Gal-3 expression paralleled with renal fibrosis, inflammation and damage, while these alterations were prevented by Gal-3 blockade. These data suggest that Gal-3 could be a new player in renal molecular, histological and functional alterations at early stages of kidney damage.

## Introduction

Chronic kidney disease (CKD) represents a significant global health problem with few therapeutic options currently known to slow its progression [1, 2]. Progressive impaired renal function results from a triad of glomerular sclerosis, tubulointerstitial fibrosis and vascular sclerosis [3]. Subclinical tubulointerstitial fibrosis may be important in the early stages of CKD [4]. The pathogenesis of renal interstitial fibrosis is driven by reorganisation of cellular interactions with the extracellular matrix, fibroblast activation, inflammation and it is characterized by an epithelial to mesenchymal transition (EMT) [5]. EMT is promoted by cytokines such as transforming growth factor beta 1 (TGF-β1) or connective tissue growth factor (CTGF) [5].

CKD is prevalent in metabolic syndrome, insulin resistance and obesity [6]. CKD can develop in obese individuals, pointing out the importance of an early detection and prevention. Obesity is associated with tubulointerstitial fibrosis which correlates with the degree of albuminuria and the progression of CKD [6]. CKD can develop in patients undergoing aortic valve replacement [7]. Aortic stenosis (AS) impairs forward blood flow from the heart, causing a chronic hypoperfusion state resulting in CKD [7]. Moreover, severe AS is one mechanism responsible for an impaired glomerular filtration rate [8].

Galectin-3 (Gal-3) is a member of β-galactoside-binding lectins which is characterized by carbohydrate recognition domain. Gal-3 has been linked to the development of renal fibrosis in animal models and it is inversely correlated with estimated glomerular filtration rate in humans [9, 10]. Elevated serum levels of Gal-3 have been associated with a higher risk of incident CKD and renal dysfunction, suggesting that Gal-3 can predict renal damage, years before CKD is detected clinically, facilitating targeted treatment and disease prevention [10]. Modified citrus pectin (MCP) (a complex water soluble indigestible polysaccharide riche in β-galactose) is a Gal-3 inhibitor that blocks the lectin’s activity. Our group has recently demonstrated that Gal-3 inhibition improves renal remodeling in hyperaldosteronism [11]. Moreover, Gal-3 inhibition is beneficial in acute kidney injury [12] and protects against hypertensive nephropathy [13].

The aim of this study was to highlight the effects of Gal-3 inhibition in early stages of mild kidney damage, using two different normotensive pathophysiological animal models: obese rats and AS rats.

## Materials and Methods

### Animals

Adult male Wistar rats weighing 150g were obtained from Harlan Ibérica. The first set of animals received either a high-fat diet (HFD, 33.5% fat; Harlan Teklad #TD.03307, MN, USA; n = 16) or a standard diet (3.5% fat; Harlan Teklad #TD.2014, MN, USA; n = 16) for 6 weeks. Half of the animals of each group received the Gal-3 activity inhibitor, modified citrus pectin, MCP (EcoNugenics, 100 mg/kg/day) in the drinking water for the same period. The second set of rats was distributed in three groups: Control rats (Control; n = 8), rats with partial occlusion of ascending aorta (AS; n = 8) and rats with partial occlusion of ascending aorta receiving the Gal-3 inhibitor MCP (AS+MCP; 100 mg/kg/day; n = 8) in the drinking water. For the occlusion of the ascending aorta, animals were anesthetized by inhalation of isoflurane. The ascending aorta was partially occluded by a hemoclip set to an outer diameter of 0.9 mm. Treatment with MCP was initiated 1 day prior to partial occlusion of ascending aorta and continued for another 6 weeks.

The investigation was performed in accordance with the Guide for Care and Use of Laboratory Animals published by the U.S. National Institutes of Health (publication no. 82–23, revised in 1996) and were approved by the local ethical committee “Comité regional Nancy-Lorraine/Nord-Est” (n° B54-547-20). Body weight was measured once a week. Food and water intake were determined throughout the experimental period. After 6 weeks of treatment, urine was collected in metabolic cages. Systolic blood pressure (SBP) was estimated before the study, at mid-study and end-of-study using a tail-cuff plethysmograph (Narco Bio-Systems). For euthanasia, rats were anesthetized i.p. with a cocktail of ketamine (Imalgene 1000) 70mg/kg and xilacine (Rompun 2%) 6mg/kg.

### Morphological and histological evaluation

Kidney tissue samples were dehydrated, embedded in paraffin and cut in 4 μμm-thick sections. Sections were stained with picrosirius red. The area of kidney tubulointerstitial fibrosis was identified as the ratio of interstitial fibrosis to the total tissue area. For each sample, 15 to 20 fields were analyzed with a 40X objective under transmitted light microscope (Leica DM 2000; Leica AG, Germany). Quantitative analysis was performed using an analysis system (Leica LAS 4.3; Leica AG, Germany).

### Real-time reverse transcription PCR

Total RNA from kidney was extracted with Trizol Reagent (Euromedex, Souffelweyersheim, France) and purified using the RNeasy kit, according to the manufacturer’s instructions (Qiagen, Hilden, Germany). First strand cDNA was synthesized according to the manufacturer’s instructions (Bio-Rad, California, USA). Quantitative PCR analysis was then performed with SYBR green PCR technology (Bio-Rad, California, USA) (S1 Table). Relative quantification was achieved with MyiQ (Bio-Rad, California, USA) software according to the manufacturer’s instructions. Data were normalized by HPRT and β-actin levels and expressed as percentage relative to controls. All PCRs were performed at least in triplicate for each experimental condition.

### ELISA

Albumin, creatinine and NGAL concentrations were measured in the plasma and urine samples by ELISA according to the manufacturer's instructions (Abcam, Cambridge, UK).

### Statistical analyses

Data are expressed as mean ± SEM. Normality of distributions was verified by means of the Kolmogorov–Smirnov test. Data were analyzed using a one-way analysis of variance, followed by a Newman-Keuls to assess specific differences among groups or conditions using GraphPad Software Inc. The predetermined significance level was P < 0.05.

## Results

### Effects of Gal-3 inhibition on renal parameters in HFD rats

To progress in the understanding of the role of Gal-3 in the renal changes associated with obesity, we used a model of diet-induced obesity previously described in which no modifications in blood pressure were observed [14] in order to avoid any potential confounding on kidney fibrosis and inflammation.

General and renal parameters of rats are presented in Table 1. The HFD induced an increase in body weight that was not modified by MCP administration (Table 1). Obese animals showed renal hypertrophy characterized by higher relative renal weight than control animals (Table 1), an effect that was not prevented with the pharmacological inhibition of Gal-3. At the end of the treatment, plasma albumin and plasma creatinine were not different in HFD rats compared with controls. By contrast, HFD rats presented increased albuminuria (Table 1). Accordingly, HFD rats exhibited enhanced albumin-creatinine ratio as compared to controls. Moreover, in HFD animals, plasma and urinary NGAL were elevated. All these parameters were reduced by MCP treatment. MCP treatment was also able to decreased plasma albumin (Table 1). No differences were found in either general or renal parameters when MCP was administered alone (data not shown).

**Table 1:** Effect of the inhibition of Gal-3 on general and renal parameters in HFD rats.

	Control	HFD	HFD+MCP
Body Weight (g)	320.4±21	372.7±21*	385.7±17*
KW/TL (g/mm)	2.22±0.05	2.49±0.08*	2.46±0.05*
SBP (mmHg)	123±3	123±3	126±2
Plasma albumin (mg/ml)	26.5±2	27.6±2	19±0.6^$^
Plasma creatinine (mg/dl)	0.44±0.03	0.33±0.02	0.39±0.03
Albumin/creatinine ratio	60.2±3	83.6±7*	48.7±4^$^
Plasma NGAL (ng/ml)	23±1	35±2*	26±3^$^
Urine albumin (μg/ml)	18.1±1	30.2±5*	21.1±3^$^
Urine NGAL (ng/ml)	84±11	118±10*	101±5 ^$^^,^*
Urine volume (ml/day)	14.7±1	8.9±1*	11.6±1^$^

HFD: high fat diet; MCP: modified citrus pectin; KW: kidney weight; TL: tibia length; SBP: systolic blood pressure; NGAL: neutrophil gelatinase-associated lipocalin. Data values represent mean±SEM of 8 animals.

* p<0.05 vs. Control group

^$^ p<0.05 *vs*. HFD group.

### Effects of Gal-3 inhibition on renal fibrosis, inflammation and damage in HFD rats

**Fig 1:**
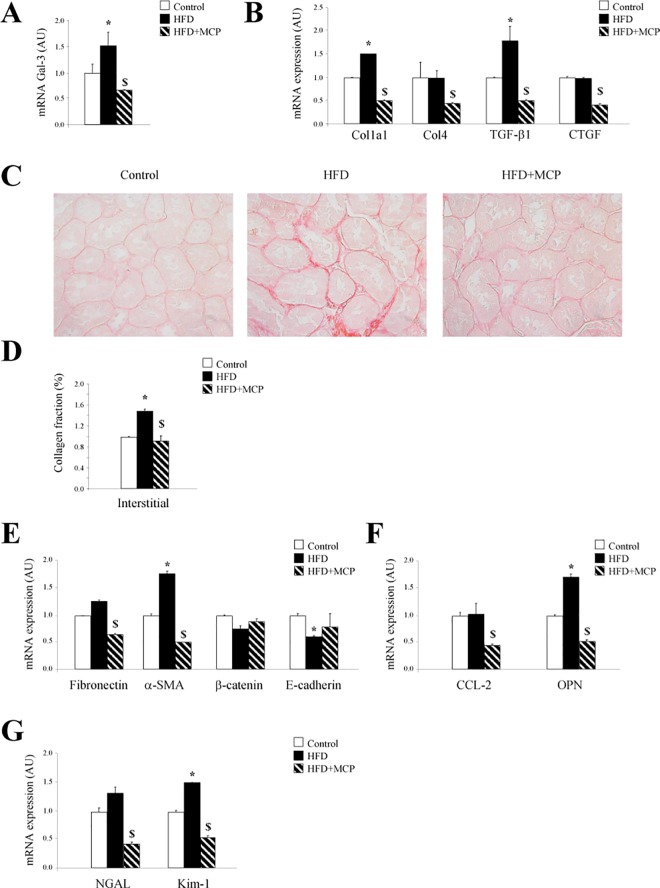
Effects of pharmacological inhibition of Gal-3 on renal damage in obese rats. Expression of mRNA of Gal-3 **(A)**. Quantification of fibrotic markers expression (**B**). Representative microphotographs of renal sections stained with Sirius red (**C**). Quantification of tubulointerstitial fibrosis (**D**). Expression of epithelial-mesenchymal transition molecules (**E**). Expression of inflammatory mediators **(F)**. Expression of kidney damage markers **(G)**. Magnification of the microphotographs 40x. Histogram bars represent the mean ± SEM of each group of animals (n ≥ 7 per group) in arbitrary units or as a percentage of staining normalized to HPRT and β-actin for cDNA. *p<0.05 vs. control group; ^$^p<0.05 vs HFD group.

The expression of Gal-3 in kidney was investigated in diet-induced obese rats. HFD rats presented increased renal Gal-3 expression (Fig 1A). The Gal-3 inhibitor MCP treatment abolished the Gal-3 increase observed in obese animals (Fig 1A).

HFD rats presented an increase in renal collagen type I and the fibrotic mediator TGF-β1 as compared with control, at mRNA levels. This increase in extracellular matrix components observed in obese animals was prevented by MCP treatment (Fig 1B). Moreover, collagen type IV and CTGF mRNA levels were lower in MCP-treated rats as compared to controls and HFD animals (Fig 1B).

The effect of Gal-3 blockade on collagen content in kidney from HFD rats is shown in Fig 1C. The increased renal interstitial collagen in HFD rats was prevented by MCP treatment (Fig 1D).

To investigate the potential involvement of Gal-3 in EMT, the expression of both markers of myofibroblats and epithelial cells was analyzed. HFD enhanced α-SMA and decreased E-cadherin levels, effects that were normalized by Gal-3 blockade (Fig 1E). Moreover, MCP treatment was able to decrease fibronectin levels (Fig 1E).

In addition, HFD rats presented renal increase in osteopontin (Fig 1F). The pharmacological inhibition of Gal-3 blocked the increase in osteopontin induced by HFD (Fig 1F). Moreover, CCL-2 levels were lower in MCP-treated rats as compared to controls and HFD animals.

Finally, the expression of kidney injury markers revealed that MCP treatment was able to decrease NGAL and Kim-1 in HFD rats (Fig 1G).

### Effects of Gal-3 inhibition on renal parameters in AS rats

Rats subjected to partial occlusion of ascending aorta presented similar body weight, renal weight and blood pressure as compared to control rats (Table 2). By contrast, MCP-treated AS rats presented a moderate decrease in relative kidney weight as compared to controls or AS animals. AS rats exhibited higher plasma and urinary NGAL as well as albuminuria as compared to Controls. MCP treatment completely restored these parameters (Table 2). Moreover, plasma albumin was decreased in MCP-treated rats as compared to AS rats.

**Table 2:** Effect of the inhibition of Gal-3 on general and renal parameters in AS rats.

	Control	AS	AS+MCP
Body Weight (g)	332±13	358±7	360±9
KW/BW (mg/g)	2.99±0.07	2.94±0.05	2.77±0.06*^$^
SBP (mmHg)	117±2	113±5	111±4
Plasma albumin (mg/ml)	21.2±2	25±2	16±0.5^$^
Plasma creatinine (mg/dl)	0.41±0.02	0.38±0.01	0.39±0.02
Albumin/creatinine ratio	51.7±5	65.8±4*	41.0±2^$^
Plasma NGAL (ng/ml)	20±1	29±2*	21±1^$^
Urine albumin (μg/ml)	20.2±1	27.1±3*	22.1±1^$^
Urine NGAL (ng/ml)	80±10	97±9*	85±7^$^
Urine volume (ml/day)	12.2±1	10±2	11±3

AS: aortic stenosis; MCP: modified citrus pectin; KW: kidney weight; BW: Body weight; SBP: systolic blood pressure; NGAL: neutrophil gelatinase-associated lipocalin. Data values represent mean±SEM of 8 animals.

* p<0.05 vs. Control group

^$^ p<0.05 *vs*. AS group.

**Fig 2:**
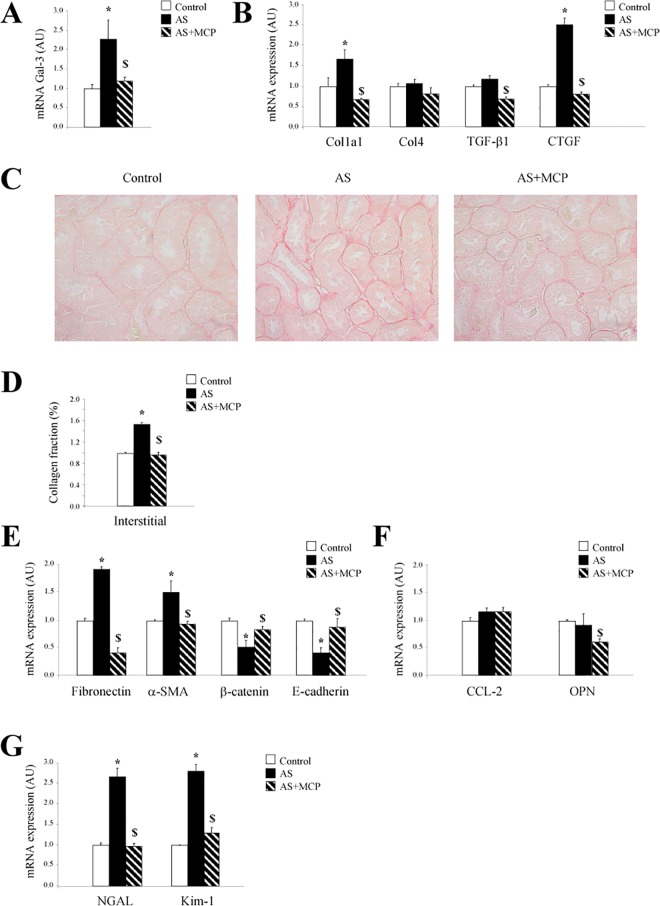
Effects of pharmacological inhibition of Gal-3 on renal damage in AS rats. Expression of Gal-3 **(A)**. Quantification of fibrotic markers expression (**B**). Representative microphotographs of renal sections stained with Sirius red (**C**). Quantification of tubulointerstitial fibrosis (**D**). Expression of epithelial-mesenchymal transition molecules (**E**). Expression of inflammatory mediators **(F)**. Expression of kidney damage markers **(G)**. Magnification of the microphotographs 40x. Histogram bars represent the mean ± SEM of each group of animals (n ≥ 7 per group) in arbitrary units or as a percentage of staining normalized to HPRT and β-actin for cDNA. *p<0.05 vs. control group; ^$^p<0.05 vs AS group.

In the kidney, AS rats presented increased Gal-3 levels, that was normalized by MCP treatment (Fig 2A).

The increase in Gal-3 levels in AS animals was accompanied by enhanced levels of renal collagen type I and CTGF mRNA levels. MCP treatment was able to normalize the fibrotic markers levels (Fig 2B). AS animals exhibited similar levels of collagen type IV and TGF-β1 expressions. However, MCP treatment reduced TGF-β1 expression in AS rats (Fig 2B).

Accordingly, the increase in tubulointerstitial fibrosis presented in AS rats was blocked by MCP treatment (Fig 2C and 2D).

Renal fibronectin and α-SMA expressions were greater in AS animals as compared to controls (Fig 2E). Complementary, AS group presented decreased renal β-catenin and E-cadherin expressions (Fig 2E). Gal-3 blockade normalized EMT markers in AS rats.

Also, AS rats treated with MCP presented decreased osteopontin levels (Fig 2F). AS animals did not present any modification in CCL-2 or osteopontin levels (Fig 2F).

The kidney injury markers NGAL and KIM-1 were enhanced in AS animals (Fig 2G). Gal-3 blockade with MCP reduced the three parameters analyzed (Fig 2G).

MCP alone did not exert modifications in any of the parameters studied (data not shown).

## Discussion

The purpose of this study was to investigate the role of Gal-3 inhibition in mild renal damage in two different physiopathological set-ups. In two normotensive animal models, obesity and AS, Gal-3 pharmacological inhibition prevented renal alterations including renal fibrosis, EMT and inflammation, all features being learnt to be associated with the progression of kidney damage. Thus, Gal-3 emerges as a new player involved in early renal alterations associated with two prevalent but very different pathological conditions such as obesity and AS.

Several studies have investigated the role of Gal-3 in acute kidney injury [15] as well as the beneficial effects of Gal-3 inhibition using MCP in experimental models of acute kidney injury [12]. MCP treatment reduced renal fibrosis, inflammation and apoptosis [12]. Besides its role in acute kidney injury, Gal-3 is also involved in the pathogenesis of CKD. In patients with heart failure, high plasma Gal-3 levels correlated with parameters associated with renal dysfunction [16]. Furthermore, Gal-3 positively correlated with increased risk of incident CKD [17]. In experimental hypertensive nephropathy, targeted inhibition of Gal-3 reduced proteinuria, improved renal function and decreased renal damage [13]. Our data are in line with these observations, showing that inhibition of Gal-3 attenuated renal structural deterioration in obese or AS rats with early renal impairment. Thus, Gal-3 blockade resulted in reduced albuminuria, tubulointerstitial fibrosis, EMT, inflammation and kidney injury markers. However, our findings are in contrast with another study showing that Gal-3 protected renal tubules against chronic injury [18]. The authors suggest that Gal-3 limited apoptosis and led to enhanced matrix remodeling and fibrosis attenuation [18]. In line with this publication, another study using Gal-3 knockout mice showed the protective role of Gal-3 in glomerular injury acting as an advanced glycation end product (AGE) receptor [19]. However, a different study using a model of unilateral ureter obstruction demonstrated that genetic disruption of Gal-3 attenuated renal fibrosis [9]. Furthermore, it has been reported that the absence of Gal-3 is related to better renal outcomes and reduced inflammation in Gal-3 knockout mice subjected to renal ischemia and reperfusion injury [20]. Moreover, our group reported that mice lacking Gal-3 are resistant to Aldosterone-induced renal fibrosis [11]. Despite the controversy about Gal-3 knockout mice and kidney injury, several studies using Gal-3 inhibitors have pointed out the protective effects of Gal-3 blockade in numerous pathological conditions such as cancer [21], cardiac fibrosis [13, 22, 23], vascular fibrosis [11], liver fibrosis [24], pulmonary fibrosis [25], adipose tissue fibrosis [26] and renal fibrosis [11–13].

CKD is a major worldwide public health concern as it can lead to progressive kidney function deterioration, substantial morbidity and increased mortality [27]. A recent phase II study comparing treatment with a Gal-3 inhibitor (GCS-100) and a placebo in patients with CKD stage 3b showed that Gal-3 inhibition improved the glomerular filtration rate (unpublished data, ClinicalTrials.gov Identifier: NCT01843790). Progressive tubulointerstitial fibrosis is considered as one of the principal hallmarks of CKD [28]. Pathogenetic factors as diverse as hypertension, proteinuria, hyperlipidemia and inflammation mediators initiate and contribute to cellular events leading to renal fibrosis [29]. The underlying cellular mechanisms include the increase in profibrotic factors, EMT and inflammation [21, 30]. We showed that MCP treatment decreased epithelial TGF-β1 and CTGF expression, two molecules that act synergistically to promote chronic fibrosis [31]. Moreover, obese and AS rats presented alterations in the expression of EMT markers, that was normalized by MCP treatment. In addition to profibrotic factors and EMT, the proinflammatory cytokines CCL-2 and osteopontin were also reduced by MCP treatment. Finally, albuminuria that may contribute *per se* to the development and progression of kidney disease by inducing tubulointerstitial inflammation, fibrosis and tubular cell injury [32] was also decreased by MCP. Furthermore, obesity and AS induced an increase in both urinary and serum NGAL, an immediate early gene increased in the most severe cases of renal injury, that were prevented by Gal-3 blockade.

In summary, Gal-3 emerges as a new factor involved in renal fibrosis, inflammation and damage in obesity and AS. The present study demonstrates the beneficial effects of Gal-3 blockade in early stages of experimental renal impairment associated with obesity and AS. Targeting Gal-3 may be an upstream therapeutic option for the treatment of kidney damage that accompanied pathological conditions such as obesity and AS.

## Supporting Information

S1 Table Primers used in rats in real time PCR analysis. (DOCX)
