# Supplementary material for: Galectin-3 Blockade Reduces Renal Fibrosis in Two Normotensive Experimental Models of Renal Damage
Source: PLoS One. 2016 Nov 9;11(11):e0166272. doi: 10.1371/journal.pone.0166272 (PMC5102450; doi:10.1371/journal.pone.0166272)
Supplement: S1 Table — (DOCX) [file pone.0166272.s001.docx]

**SUPPLEMENTAL TABLES**

**Table S1: Primers used in rats in real time PCR analysis**

| Gene | Primer | Sequence (5´to 3´) |
| --- | --- | --- |
| Gal-3 | Forward | AGCCCAACGCAAACAGTATC |
|  | Reverse | GGCTTCAACCAGGACCTGTA |
| Col1a1 | Forward | GCCTCCCAGAACATCACCTA |
|  | Reverse | ATGTCTGTCTTGCCCCAAGT |
| Fibronectin | Forward | GGGGTCACGTACCTCTTCAA |
|  | Reverse | TGGAGGTTAGTGGGAGCATC |
| TGF-β | Forward | CAGAAGTTGGCATGGTAGCC |
|  | Reverse | TGCTTCAGCTCCACAGAGAA |
| CTGF | Forward | GAGTCGTCTCTGCATGGTCA |
|  | Reverse | CCACAGAACTTAGCCCGGTA |
| OPN | Forward | ATGAGACTGGCAGTGGTT |
|  | Reverse | GCTTTCATTGGAGTTGCT |
| CCL2 | Forward | TTCCTTATTGGGGTCAGCAC |
|  | Reverse | CAGTTAATGCCCCACTCAC |
| Col-IV | Forward | GCCAAGTGTGCATGAGAAGA |
|  | Reverse | AGCGGGGTGTGTTAGTTACG |
| α-SMA | Forward | GAAGGAATAGCCACGCTCAG |
|  | Reverse | TGTGCTGGACTCTGGAGATG |
| β-Catenin | Forward | GCCAGTGGATTCCGTACTGT |
|  | Reverse | GAGCTTGCTTTCCTGATTGC |
| E-Cadherin | Forward | GGGTTGTCTCAGCCAATGTT |
|  | Reverse | CACCAACACACCCAGCATAG |
| NGAL | Forward | TCAGCCTGTACGGAAGAACC |
|  | Reverse | GGTGGGAACAGAGAAAACGA |
| KIM-1 | Forward | AGAGAGAGCAGGACACAGGCTT |
|  | Reverse | ACCCGTGGTAGTCCCAAACA |
| HPRT | Forward | AGGACCTCTCGAAGTGT |
|  | Reverse | ATTCAAATCCCTGAAGTACTCAT |

| β-Actin | Foward | CCTCTATGCCAACACAGTGCTGTCT |
| --- | --- | --- |
|  | Reverse | GCTCAGGAGGAGCAATGATCTTGA |
